# Finite-Depth Interfacial Coupling in Metal/Topological Insulator van der Waals Heterostructures

**DOI:** 10.1021/acs.nanolett.6c04373

**Published:** 2026-11-02

**Authors:** Yin-Song Liao, Hsin-Hsien Chan, Ruei-Yu Wang, Han-Wei Tsai, Hsun-Ting Hsieh, Cheng-Maw Cheng, Jyh-Pin Chou, Meng-Kai Lin

**Affiliations:** † Graduate School of Advanced Technology, 33561 National Taiwan University, Taipei 106319, Taiwan; ‡ Department of Physics, National Central University, Taoyuan 32001, Taiwan; § 57815 National Synchrotron Radiation Research Center, Hsinchu 30076, Taiwan

**Keywords:** Transition metal dichalcogenides, Topological insulators, Moiré modulations, Ultrathin films, Emergent bands, van der Waals heterostructures

## Abstract

Metal/topological insulator van der Waals (vdW) heterostructures offer a platform for engineering emergent quantum states beyond isolated layers. While proximity effects in covalently bonded systems are well studied, coupling depth across vdW interfaces remains unclear. Using angle-resolved photoemission spectroscopy and first-principles calculations, we investigate the thickness dependence of TiTe_2_/Bi_2_Te_3_ electronic states. Despite nominally weak vdW bonding, the buried interface induces emergent bands and splitting near a band crossing, consistent with hybridization. Interface-induced features persist in the bilayer, while band evolution nearly saturates in thicker films. Even at four layers, the electronic structure does not simply recover to the intrinsic TiTe_2_ limit, consistent with finite-depth coupling that substantially reshapes the overlayer bands. Charge redistribution and a moiré-modulated interfacial potential contribute to this reconstruction. Finite-depth coupling may be a hallmark of strongly coupled vdW heterostructures and guide topological and quantum electronic device design.

## Graphical abstract

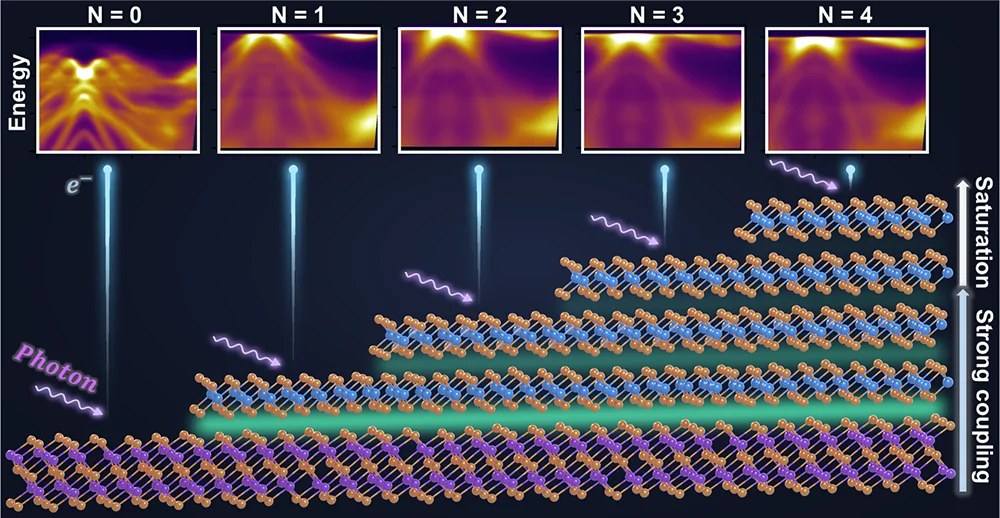

Interfacial coupling in van der Waals (vdW) heterostructures has attracted enormous attention because weak interlayer bonding enables atomically sharp interfaces while still allowing strong electronic interactions across the interface.^1–18^ Unlike conventional epitaxial heterostructures, vdW systems provide a unique platform for engineering interfacial quantum states through charge transfer, orbital hybridization, lattice mismatch, and symmetry breaking without the constraint of strict lattice matching.^1–18^ Such interfacial coupling has been shown to induce a variety of emergent phenomena, including correlated insulating phases,^1,2^ magnetism,^3–5^ and nontrivial topological states.^6–8^ In many layered chalcogenide systems, interface interactions can dramatically reconstruct the electronic structure, leading to emergent bands,^9–13^ hybridized gap openings,^9–11^ and quasiparticle renormalization even when the constituent materials preserve their individual crystal structures.^9–18^ These findings demonstrate that vdW interfaces are not merely passive stacking boundaries, but active electronic platforms capable of generating new quantum phases.

Among these systems, heterostructures composed of metallic overlayers and topological insulators (TIs) provide a particularly important platform for understanding how topological and metallic states interact across buried interfaces. The topological surface states (TSSs) of TIs, protected by time-reversal symmetry and characterized by spin-momentum locking, can strongly modify the electronic structure of adjacent metallic layers through orbital hybridization and interfacial charge transfer.^14–16^ However, a central question remains unresolved: does the influence of a buried vdW interface remain localized at the first contact layer, or can it propagate vertically and impose a finite-depth electronic boundary condition on the overlayer? In some systems, such as Pb/TlBiSe_2_, the TSSs were reported to extend into the metallic overlayer through quantum well resonance.^14^ However, whether such long-range propagation of topological surface states is a universal feature of metal/TI vdW heterostructures remains unclear. In most experimentally studied systems, strong interfacial coupling is more commonly reflected by interfacial hybridization, emergent bands, and substantial electronic renormalization rather than direct transfer of topological surface states into the metallic overlayer.^12,13,17^ Determining the spatial extent of such coupling is therefore essential for understanding whether vdW heterostructures should be described within a weak-coupling picture or as strongly coupled emergent electronic systems.

Transition metal dichalcogenides (TMDCs) and related layered semimetals provide an ideal platform for addressing this problem because their orbital character and thickness-dependent electronic structures are highly sensitive to interfacial interactions.^19–22^ TiTe_2_, a semimetallic layered compound with strong Ti-*d* and Te-*p* orbital contributions near the Fermi level,^23–25^ is particularly suitable for investigating how metallic states evolve when coupled to a topological substrate. Bi_2_Te_3_, on the other hand, is a prototypical TI with well-defined TSSs and strong spin–orbit coupling.^26–28^ The TiTe_2_/Bi_2_Te_3_ heterostructure therefore serves as an ideal model system for exploring how interfacial hybridization, charge transfer, and electronic reconstruction propagate across a metal/TI vdW boundary.

In this work, we present a comprehensive study of 1–4 trilayer (TL) TiTe_2_ films grown on 7 quintuple-layer (QL) Bi_2_Te_3_ by combining angle-resolved photoemission spectroscopy (ARPES) and first-principles calculations. Experimental and computational details are provided in the Supporting Information. We systematically investigate the thickness-dependent evolution of the electronic structure and reveal unexpectedly strong interfacial coupling despite the nominally weak vdW interface. Pronounced emergent bands and hybridization gap openings are observed, indicating substantial orbital mixing between Ti-*d* and Te-*p* (contributed from Bi_2_Te_3_) states across the interface. These hybridized states extend to at least the second TiTe_2_ layer, accompanied by significant interfacial charge transfer and strong band renormalization. Notably, the electronic structure does not recover to the intrinsic TiTe_2_ limit even when the film thickness increases to four layers, demonstrating that the buried interface imposes a finite-depth electronic boundary condition rather than a localized perturbation. Our results establish finite-depth interfacial coupling as a defining characteristic of strongly coupled metal/TI vdW heterostructures and highlight its importance for designing topological and quantum electronic devices.

To establish the spectroscopic signatures of interfacial electronic reconstruction, we first compare the electronic structures of Bi_2_Te_3_ and TiTe_2_ films grown on bilayer-graphene-terminated 6H-SiC(0001) (BLG, for short) with those of 1–2 TL TiTe_2_/Bi_2_Te_3_ heterostructures. Figure 1(a) presents the ARPES map of 7-QL Bi_2_Te_3_/BLG measured along the $\overset{®}{\Gamma M}$ direction. The 7-QL thickness was chosen to ensure that the Bi_2_Te_3_ film reaches the bulk-like limit, where quantum confinement effects are largely suppressed and the intrinsic TSS is represented.^26–28^ A well-defined Dirac cone is clearly resolved near the Γ̅, with the Dirac point located approximately 0.2 eV below the Fermi level, consistent with previous reports.^26–28^ The sharp TSS confirms the high crystalline quality of the Bi_2_Te_3_ film and provides a reliable topological substrate for constructing the metal/TI heterostructure.

**Figure 1:**
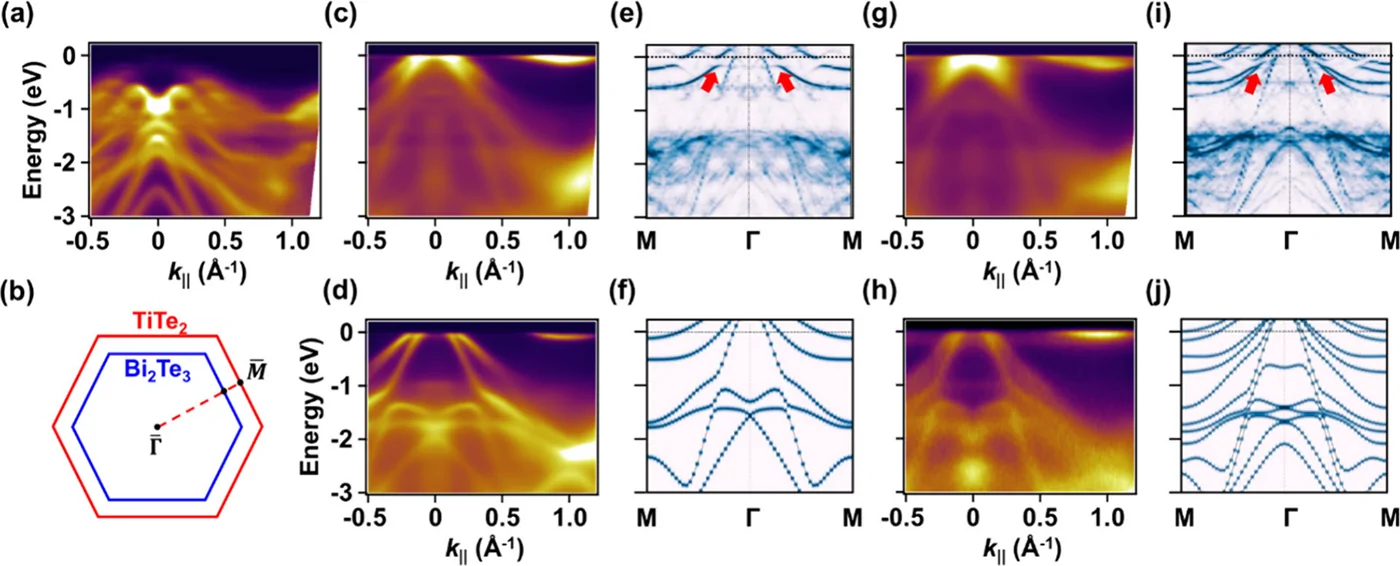
Band structure characterization of TiTe_2_/Bi_2_Te_3_ heterostructures. (a) ARPES map of 7-QL Bi_2_Te_3_ grown on BLG measured along the $\overset{®}{\Gamma M}$ direction. (b) Schematic illustration of the Brillouin zones of the TiTe_2_ overlayer and the Bi_2_Te_3_ substrate, indicating the parallel epitaxial alignment of the two lattices. (c, d) ARPES maps of 1-TL TiTe_2_ grown on Bi_2_Te_3_ and 1-TL TiTe_2_ grown on BLG, respectively. (e, f) Corresponding calculated band structures of the heterostructure and freestanding 1-TL TiTe_2_ shown in (c) and (d). (g, h) ARPES maps of 2-TL TiTe_2_ grown on Bi_2_Te_3_ and 2-TL TiTe_2_ grown on BLG, respectively. (i, j) Corresponding calculated band structures of the heterostructure and freestanding 2-TL TiTe_2_ shown in (g) and (h). All band structures are taken along the $\overset{®}{\Gamma M}$ direction. The red arrows in Figures (e) and (i) point out the avoided-crossing regions.

Figure 1(b) shows the Brillouin zones and confirms the parallel in-plane orientation of TiTe_2_ and Bi_2_Te_3_. The alignment is supported by RHEED and ARPES measurements along the $\overset{®}{\Gamma K}$ direction (Figures S1 and S2), while the corresponding layer-projected calculation is shown in Figure S3 for comparison.

Figure 1(c) shows the ARPES map of 1-TL TiTe_2_/Bi_2_Te_3_ heterostructure, while Figure 1(d) presents 1-TL TiTe_2_/BLG for direct comparison. The heterostructure exhibits a band structure that differs substantially from 1-TL TiTe_2_/BLG and cannot be described as a simple superposition of isolated TiTe_2_ and Bi_2_Te_3_ bands. Instead, pronounced interface-induced electronic reconstruction is observed. Emergent bands appear at approximately −0.91, −0.67, −0.22, and −0.03 eV. More importantly, clear hybridization gaps are observed where the TiTe_2_- and Bi_2_Te_3_-derived bands intersect, providing direct evidence of coherent orbital hybridization across the vdW interface. To elucidate the origin of the hybridization, we performed orbital-projected band structure calculations for freestanding 1-TL TiTe_2_ (Figure S4), freestanding 7-QL Bi_2_Te_3_ (Figure S5), and heterostructure of 1-TL TiTe_2_ on 7-QL Bi_2_Te_3_ (Figure S6). The results indicate that these emergent states originate from hybridization between Ti-*d* and Te-*p* orbitals contributed by both TiTe_2_ and Bi_2_Te_3_. This strong *d*–*p* orbital mixing drives hybridization gap opening and substantial quasiparticle reconstruction near the Fermi level.

In addition to hybridization, the heterostructure exhibits a rigid energy shift relative to 1-TL TiTe_2_. Compared with 1-TL TiTe_2_, the valence-band maximum shifts toward lower binding energy by approximately 0.1 eV, indicating electron doping of the TiTe_2_ overlayer. This shift is consistent with interfacial charge transfer from Bi_2_Te_3_ to TiTe_2_, as further supported by charge density difference (CDD) calculations discussed below in Figure 4. The corresponding calculated band structures of 1-TL TiTe_2_ on 7-QL Bi_2_Te_3_ and freestanding 1-TL TiTe_2_ are shown in Figures 1(e,f). The calculations capture the overall band connectivity and interface-induced reconstruction, although quantitative differences in binding energies, spectral visibility, and several small-gap features remain between theory and experiment. Such differences are expected because Kohn–Sham eigenvalues are not, in general, quantitatively identical to quasiparticle excitation energies, while ARPES spectral intensity additionally depends on photoemission matrix elements and final-state effects.^29,30^ For 1T-TiTe_2_, ARPES spectral intensities are strongly influenced by photoemission matrix-element effects.^31^ Similar quantitative deviations between ARPES and density-functional band structures have also been reported for 1T-TiTe_2_.^32^ Accordingly, the calculations are used primarily to identify the orbital/layer character and hybridization pathways of the reconstructed states rather than to reproduce the ARPES spectral intensity on a band-by-band basis.

To examine whether the interfacial electronic reconstruction extends beyond the first contact layer, we compare the 2-TL TiTe_2_/Bi_2_Te_3_ heterostructure with 2-TL TiTe_2_/BLG, as shown in Figures 1(g,h). Although the strongest reconstruction is expected in the TiTe_2_ layer directly contacting Bi_2_Te_3_, the heterostructure still exhibits interface-related features and incomplete recovery of the intrinsic bilayer dispersion. Because ultraviolet ARPES is highly surface sensitive, these features cannot be attributed solely to direct photoemission from the buried interface. The calculated layer-projected band structure shows a corresponding avoided-crossing-like feature in this momentum region (Figure 1(i) and Figure S7), although its energy position differs slightly from experiment. In contrast, comparable momentum-localized splitting is absent in the 2-TL TiTe_2_/BLG spectrum (Figure 1(h)). These combined results indicate that the upper TiTe_2_ layer remains modified by the underlying Bi_2_Te_3_ substrate, implying the interfacial coupling extends beyond the first contact layer.

To clarify the spatial extent of the interfacial coupling, we performed thickness-dependent ARPES measurements on *N*-TL TiTe_2_ films grown on Bi_2_Te_3_, with *N* ranging from 0 to 4. The ARPES and second-derivative maps are shown in Figures 2(a,b). The most pronounced evolution occurs between *N* = 1 and *N* = 2. In particular, the second-derivative maps reveal an interface-related splitting of the second valence band in both 1-TL and 2-TL TiTe_2_/Bi_2_Te_3_, whereas this feature is absent in the corresponding TiTe_2_/BLG films. The persistence of the splitting in the 2-TL film indicates that the interfacial influence extends beyond the first contact layer. Momentum-dependent EDCs from the original ARPES data show that the feature evolves from a single peak into two distinguishable peaks and then merges back into a single peak as *k*_||_ increases (see Figure S8). This finite-momentum splitting and recombination is consistent with an avoided-crossing-like interaction between dispersive branches rather than simply an additional thickness-derived subband. Detailed EDC extraction and fitting procedures are provided in the Supporting Information.

**Figure 2:**
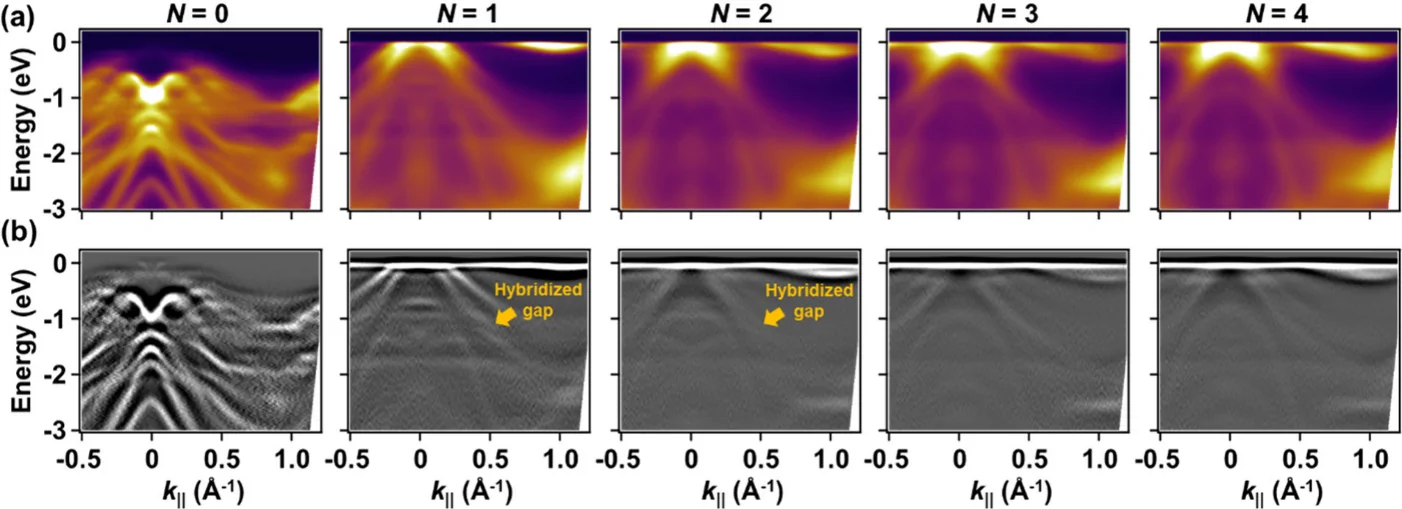
Thickness-dependent evolution of the electronic structure in TiTe_2_/Bi_2_Te_3_ heterostructures. (a) ARPES maps of *N*-TL TiTe_2_ films (*N* = 0–4) grown on Bi_2_Te_3_ measured along the $\overset{®}{\Gamma M}$ direction, where *N* = 0 corresponds to 7-QL Bi_2_Te_3_ grown on BLG. (b) Corresponding second-derivative ARPES maps of (a).

For *N* > 2, only weak additional band evolution is observed. The electronic structures of the 3-TL and 4-TL films are nearly identical to the 2-TL spectrum, even though few-layer TiTe_2_ remains distinct from bulk TiTe_2_. Previous ARPES measurements of intrinsic TiTe_2_ revealed continued layer-dependent evolution of the valence-band subbands, including band doubling in the bilayer, additional subband features in the trilayer, and a more complex bulk continuum associated with dispersion along the layer-stacking direction.^24^ Bulk TiTe_2_ also exhibits a genuinely three-dimensional electronic structure with *k*_*z*_-dependent band inversion and orbital-character changes that are absent in the monolayer limit.^23^ Thus, the similarity between the 3-TL and 4-TL spectra in the present heterostructure should not be interpreted as complete recovery to the bulk limit. Instead, their near-saturation indicates that the heterostructure approaches an interface-renormalized finite-layer electronic structure. The persistence of interface-related features up to 2-TL and the weak evolution at larger thickness establish a finite coupling depth in which interface-induced reconstruction dominates over the intrinsic thickness evolution of TiTe_2_.

Following the thickness-dependent ARPES results, the interfacial reconstruction retains beyond the first TiTe_2_ layer and becomes nearly saturated at *N* = 2, we next examine the origin and spatial character of the emergent bands. In particular, we aim to determine whether these additional band features are localized at the buried interface or represent hybridized states with finite penetration into the TiTe_2_ overlayer.

Figure 3(a) shows a zoomed-in ARPES map of 7-QL Bi_2_Te_3_ grown on BLG near the Γ̅, while Figure 3(b) presents the corresponding second-derivative map. The Dirac cone is clearly resolved with the characteristic linear dispersion of the TSS, serving as the reference electronic structure of the Bi_2_Te_3_ substrate. No additional flat or weakly dispersive features are observed within this energy window, indicating that the emergent bands discussed below do not originate from intrinsic Bi_2_Te_3_ band features.

**Figure 3:**
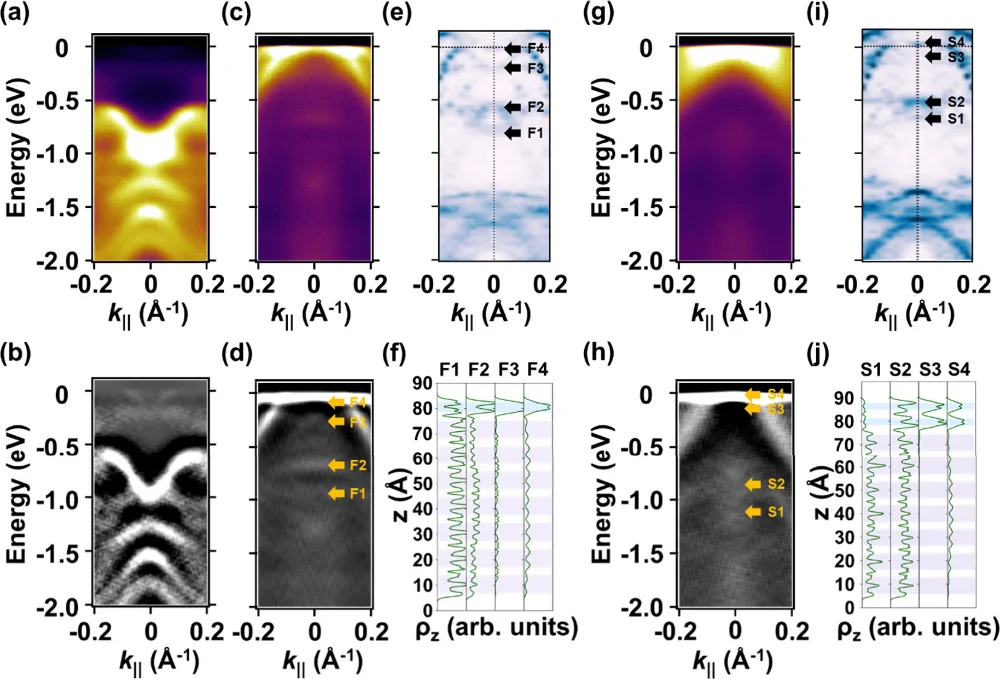
Emergent bands and nonlocal interfacial hybridization in TiTe_2_/Bi_2_Te_3_ heterostructures. (a, b) Zoomed-in ARPES map and the corresponding second-derivative map of 7-QL Bi_2_Te_3_ grown on BLG near the Γ̅ point. (c–e) Zoomed-in ARPES map, corresponding second-derivative map, and calculated band structure of 1-TL TiTe_2_ on 7-QL Bi_2_Te_3_, respectively. (f) Layer-resolved DOS distribution of the emergent bands F1–F4. (g–i) Zoomed-in ARPES map, corresponding second-derivative map, and calculated band structure of 2-TL TiTe_2_ on 7-QL Bi_2_Te_3_, respectively. (j) Layer-resolved DOS distribution of the emergent bands S1–S4.

Figure 3(c) presents the zoomed-in ARPES map of the 1-TL TiTe_2_/Bi_2_Te_3_ heterostructure, and Figure 3(d) shows the corresponding second-derivative map. Several additional band features, labeled F1–F4, emerge near the Γ̅. These bands are absent in both 7-QL Bi_2_Te_3_/BLG and 1-TL TiTe_2_/BLG, ruling out a simple superposition of the constituent band structures. The near-Γ̅ F1–F4 bands should be distinguished from the finite-*k*_||_ avoided-crossing-like feature discussed in Figure 2. The latter provides evidence for momentum-dependent interfacial band hybridization, whereas the appearance of F1–F4 and their absence in the reference films indicate additional interface-induced electronic states. The calculated band structure in Figure 3(e) reproduces the main dispersions and approximate energy positions of the F1–F4 features. As noted above, quantitative differences between theory and experiment are expected; nevertheless, their overall agreement supports the assignment of these features as heterostructure-induced states rather than extrinsic spectral artifacts. To further determine the spatial character of the emergent states, we analyze the layer-resolved DOS associated with F1–F4 in Figure 3(f). F1 and F2 have substantial weight across the TiTe_2_ overlayer and the upper Bi_2_Te_3_ layers, whereas F3 and F4 are more strongly concentrated within TiTe_2_. These results indicate that the heterostructure supports multiple hybridization channels with different spatial extents.

A similar behavior is observed for the 2-TL TiTe_2_/Bi_2_Te_3_ heterostructure (Figures 3(g,h)). Several emergent bands, labeled S1–S4, remain visible even though the upper TiTe_2_ layer is not directly in contact with Bi_2_Te_3_. To identify these states, we extracted an EDC at the Γ̅ from the original ARPES data and performed phenomenological fitting (Figure S9). The fitted peaks are located at approximately −1.10, −0.87, −0.23, and −0.01 eV, corresponding to S1, S2, S3, and S4, respectively. The calculated band structure in Figure 3(i) reproduces the main spectral features. The layer-resolved profiles in Figure 3(j) show substantial Bi_2_Te_3_ weight for S1, whereas S2 extends across both TiTe_2_ layers and into Bi_2_Te_3_. S3 and S4 are concentrated mainly within the TiTe_2_ overlayer. These results indicate that the finite-depth coupling is carried primarily by selected hybridized states rather than by a uniform modification of all overlayer bands.

Orbital-projected calculations shown in Figures S6, S10, and S11 indicate that the emergent bands arise from multiple TiTe_2_–Bi_2_Te_3_ hybridization channels. F1/F2 and S1/S2 contain substantial *p*_*x*_ and *p*_*y*_ contributions from both materials, whereas F3/F4 and S3/S4 have stronger Ti 3*d* and Bi_2_Te_3_ *p*_*z*_ character. Selected F1/F2 and S1/S2 states also retain appreciable weight within the Bi_2_Te_3_ slab, indicating contributions from bulk-like or subsurface Bi_2_Te_3_ states. The calculated and experimental labels therefore denote corresponding dispersive features rather than exact energy-matched eigenstates.

It is important to distinguish the spatial extent of the interface-induced electronic reconstruction from the spatial extent of the Bi_2_Te_3_ TSS itself. In the present heterostructure, the calculations indicate that the TSS remains primarily localized near the buried interface and does not propagate throughout the metallic TiTe_2_ overlayer. We therefore do not attribute all emergent bands or hybridization-induced gaps directly to the TSS. Instead, these features arise from interfacial orbital hybridization and charge redistribution involving TiTe_2_ and Bi_2_Te_3_-derived states, resulting in hybridized states with different spatial characters. Some of these states extend across the interface and into the upper TiTe_2_ layer, whereas others remain predominantly TiTe_2_-like. Thus, the finite-depth coupling reported here refers to the spatial range over which the buried interface modifies the electronic structure of the overlayer, rather than to the propagation of a pure TSS through TiTe_2_. A TSS localized at the buried interface can still form part of the interfacial electronic environment and influence nearby metallic states through local hybridization and the interface-induced potential. The experimentally observed finite-depth reconstruction is therefore carried by the resulting hybridized states and charge redistribution, rather than by the spatial extension of the TSS itself.

The spatially extended emergent states provide an explanation for the saturation behavior observed at *N* = 2 in Figure 2. The strongest electronic renormalization is not associated with atomically localized interface states with extremely short decay lengths, but with hybridized resonances that penetrate into the TiTe_2_ overlayer over a finite depth. Once the TiTe_2_ thickness exceeds this characteristic coupling length, the heterostructure approaches an interface-renormalized electronic structure, and further thickness increase produces only weak additional spectral evolution. In this sense, the buried Bi_2_Te_3_ interface acts not merely as a perturbative substrate, but as an active electronic boundary condition that reshapes the low-energy electronic structure of the TiTe_2_ overlayer.

To further understand the origin of the finite coupling depth, we examine the spatial charge redistribution in a 3-TL TiTe_2_/7-QL Bi_2_Te_3_ heterostructure using CDD analysis. The relaxed structure is shown in Figure S12. We compare the charge redistribution at the buried TiTe_2_/Bi_2_Te_3_ interface and at the two upper TiTe_2_/TiTe_2_ interfaces as shown in Figure 4.

**Figure 4:**
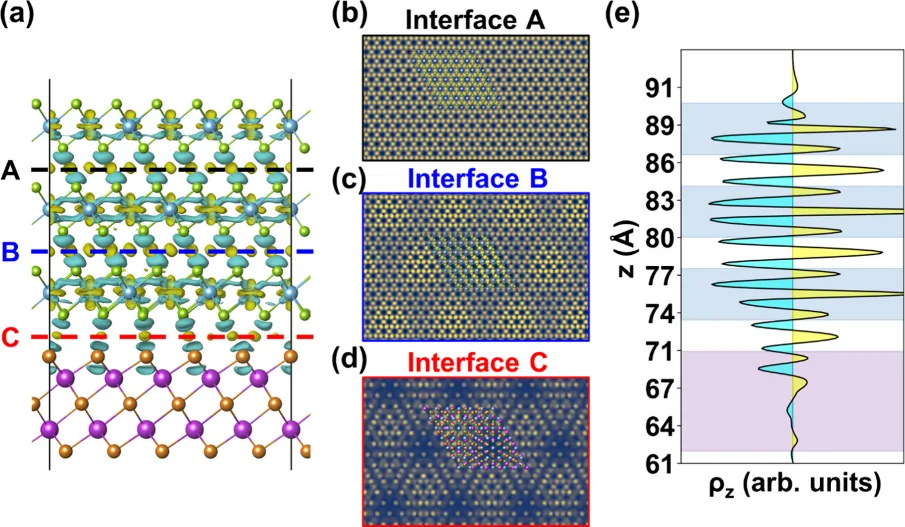
Spatial charge redistribution and moiré-driven interfacial modulation in 3-TL-TiTe_**2**_/7-QL-Bi_2_Te_3_ heterostructures. (a) Three-dimensional CDD distribution of 3-TL TiTe_2_ on 7-QL Bi_2_Te_3_. For clarity, only the topmost QL of Bi_2_Te_3_ is shown. The dashed lines labeled A, B, and C indicate the interfaces used to extract the CDD contour maps. (b–d) In-plane CDD contour maps taken at interface A, B, and C, respectively. (e) In-plane averaged CDD along the out-of-plane direction. The position of TiTe_2_ and Bi_2_Te_3_ are represented in shallow blue and purple region. In both (a) and (e), the yellow and cyan regions correspond to the charge accumulation and depletion, respectively.

Figure 4(a) shows the calculated spatial charge redistribution for 3-TL TiTe_2_ on Bi_2_Te_3_. At Interface C, corresponding to the buried TiTe_2_/Bi_2_Te_3_ boundary, the CDD contour exhibits a pronounced moiré pattern. A weaker residual modulation remains at Interface B, the TiTe_2_/TiTe_2_ interface directly above the substrate. In contrast, the charge redistribution at Interface A, farther from the substrate, is nearly uniform and no discernible moiré modulation is observed. This C-to-B-to-A evolution indicates that the moiré-modulated perturbation generated at the buried interface is transmitted into the second TiTe_2_ layer and strongly attenuated above it, consistent with the weak additional band evolution observed for *N* > 2. Additional details of the interface-dependent CDD patterns are provided in the Supporting Information.

The moiré-induced structural relaxation and interfacial hybridization should not be regarded as mutually exclusive mechanisms; rather, the spatially varying local registry modulates the orbital coupling across the TiTe_2_/Bi_2_Te_3_ interface. The structural relaxation further supports this finite-depth picture. As shown in the side-view atomic structure in Figure S12, the TiTe_2_ layer directly contacting Bi_2_Te_3_ exhibits noticeable buckling, consistent with the spatially anisotropic interaction produced by the moiré-modulated heterointerface. In particular, the Te atoms in the first TiTe_2_ layer show pronounced out-of-plane distortion, reflecting the strong spatial variation of the interfacial potential. In the second TiTe_2_ layer, only the lower Te sublayer exhibits a weak residual distortion, while the upper Te sublayer remains nearly flat. This partial structural response indicates that the interface-induced modulation propagates only weakly beyond the first TiTe_2_ layer and becomes strongly attenuated within approximately one to two layers.

Figure 4(e) presents the planar-averaged CDD along the out-of-plane direction, derived from the spatial charge redistribution shown in Figure 4(a). The magnitude of the planar-averaged CDD reflects the overall charge redistribution upon heterostructure formation and should not be interpreted as a direct quantitative measure of orbital-hybridization strength. The profile indicates that the charge redistribution is not confined to the three interfacial regions, but also involves intralayer charge rearrangement within the TiTe_2_ layers. In particular, the spatial CDD analysis in Figure 4(a) shows electron accumulation around Ti sites with a *d*_z2_-like distribution, while the planar-averaged profile in Figure 4(e) confirms the finite vertical extent of this redistribution. To further quantify the layer dependence of the substrate-induced electronic reconstruction, we compared the layer-resolved PDOS of supported 3-TL TiTe_2_ with that of the corresponding freestanding 3-TL TiTe_2_ reference. A normalized integrated PDOS difference was evaluated for the occupied states within 0.5 eV below the Fermi level, as described in the Supporting Information. The resulting values decrease from 0.1278 for the TiTe_2_ layer adjacent to Bi_2_Te_3_ to 0.1217 for the middle layer and 0.0888 for the outermost layer. This systematic reduction provides a quantitative measure of the attenuation of the near-*E*_F_ electronic perturbation away from the buried interface. Together with the orbital-projected band analysis for *N*-TL TiTe_2_ on 7-QL Bi_2_Te_3_ (Figure S6 and S10-11), this behavior is consistent with Ti-*d*/Te-*p* hybridization and supports the picture of interface-induced electronic reconstruction extending into the TiTe_2_ overlayer.

In this work, we investigate finite-depth interfacial coupling in a metal/TI vdW heterostructure composed of TiTe_2_/Bi_2_Te_3_. By combining ARPES measurements with first-principles calculations, we reveal strong interfacial electronic reconstruction driven by interfacial charge transfer and orbital hybridization across the buried vdW interface. This coupling produces a strongly charge-polarized interface, induces hybridization-related band splitting, and causes substantial band renormalization that extends beyond the first contact layer into at least the second TiTe_2_ layer, thereby defining a finite interfacial coupling depth.

Our layer-dependent band characterization further shows that the electronic structure does not recover to the intrinsic TiTe_2_ limit even when the film thickness increases to four layers. Instead, the thickness evolution rapidly saturates after the second TiTe_2_ layer, indicating that the overlayer approaches an interface-renormalized electronic state rather than the intrinsic TiTe_2_ limit. Together with CDD analysis, these results suggest that moiré-modulated charge redistribution at the TiTe_2_/Bi_2_Te_3_ interface is transmitted into the overlayer through interlayer coupling and becomes strongly attenuated beyond approximately two TiTe_2_ layers.

Importantly, the finite-depth coupling identified here should not be interpreted as the propagation of the Bi_2_Te_3_ TSS throughout the TiTe_2_ overlayer, but rather as the finite-depth electronic reconstruction carried by interfacial hybridized states and charge redistribution. Based on these observations, we propose that vdW heterostructures can be broadly discussed in terms of weak and strong interfacial-coupling regimes. In a weak-coupling picture, often expected for weakly interacting semiconducting vdW interfaces, the buried interface acts mainly as a localized perturbation, and the overlayer electronic structure progressively approaches that of the intrinsic material with increasing thickness. In contrast, in a strong-coupling regime, which may arise in metallic systems or small-gap heterostructures, interfacial reconstruction can remain significant over a finite depth, producing persistent deviations from the intrinsic band structure. This distinction is important for the design of two-dimensional quantum and electronic devices. Our results show that strongly coupled vdW heterostructures cannot always be understood within a simple pristine-layer picture. Instead, finite-depth interfacial band renormalization must be considered when evaluating their electronic properties, possible topological proximity effects, and device functionality.

## Supplementary Material



## Notes

Additional figures and discussion as described in the text (PDF)
