# Supplementary material for: Finite-Depth Interfacial Coupling in Metal/Topological Insulator van der Waals Heterostructures
Source: Nano Lett. 2026 Nov 2;26(44):15440–6. doi: 10.1021/acs.nanolett.6c04373 (PMC13761692; doi:10.1021/acs.nanolett.6c04373)
Supplement: Supplementary file 1 [file nl6c04373_si_001.pdf]

Supporting Information for "Finite-Depth Interfacial Coupling in Metal/Topological Insulator van der Waals Heterostructures"

Yin-Song Liao[1,†], Hsin-Hsien Chan[2,†], Ruei-Yu Wang[2,3] , Han-Wei Tsai[2], Hsun-Ting Hsieh[2], Cheng-Maw Cheng[3], Jyh-Pin Chou[1,*], and Meng-Kai Lin[2,*]

[1]Graduate School of Advanced Technology, National Taiwan University, Taipei 106319, Taiwan

[2]Department of Physics, National Central University, Taoyuan 32001, Taiwan

[3]National Synchrotron Radiation Research Center, Hsinchu 30076, Taiwan

Corresponding Authors

[*]Meng-Kai Lin, Email: mklin@office365.ncu.edu.tw

[*]Jyh-Pin Chou, Email: jpchou@ntu.edu.tw

Additional author note.

[†]Equal contribution

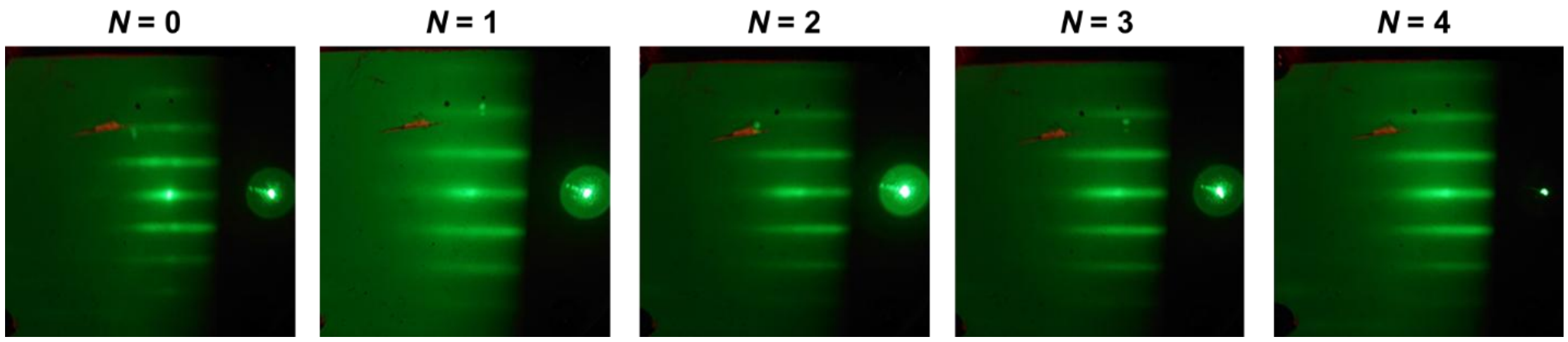

Figure S1. RHEED patterns for *N*-layer $TiTe_2$ on 7-QL Bi_2Te_3.

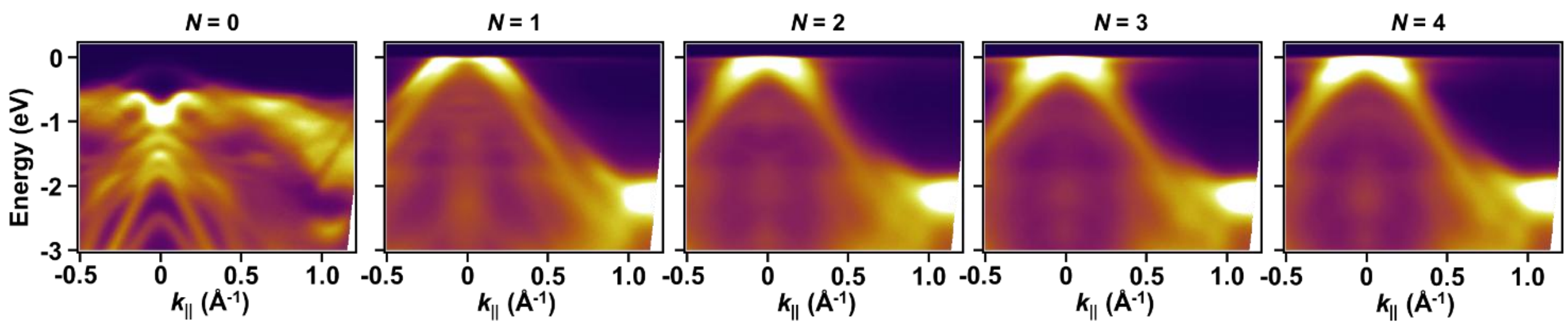

Figure S2. ARPES maps taken along the $\overline{\Gamma K}$ direction for *N*-layer $TiTe_2$ grown on 7-QL Bi_2Te_3.

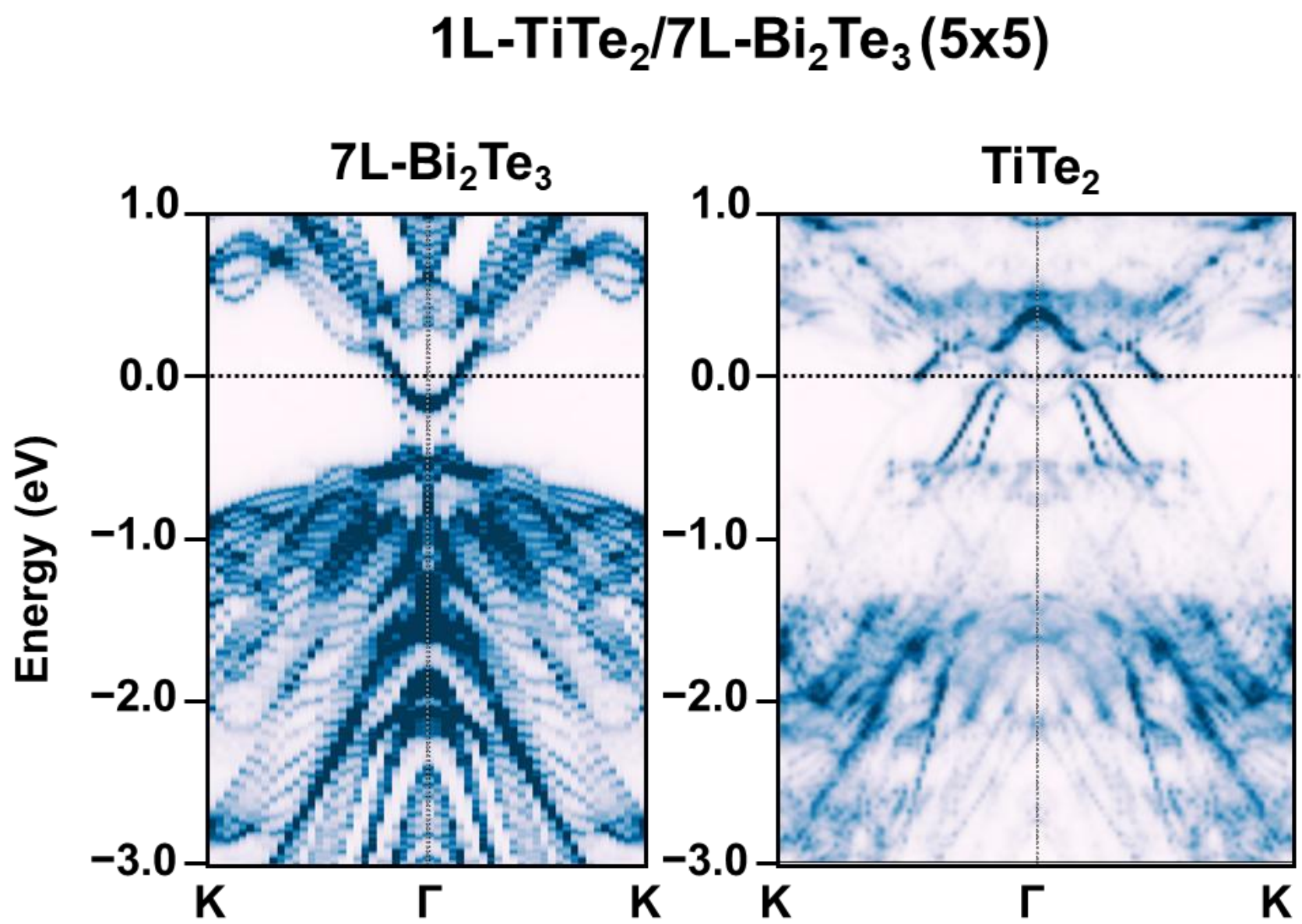

Figure S3. Layer-projected band structure for 1-TL $TiTe_2$ on 7-QL Bi_2Te_3.

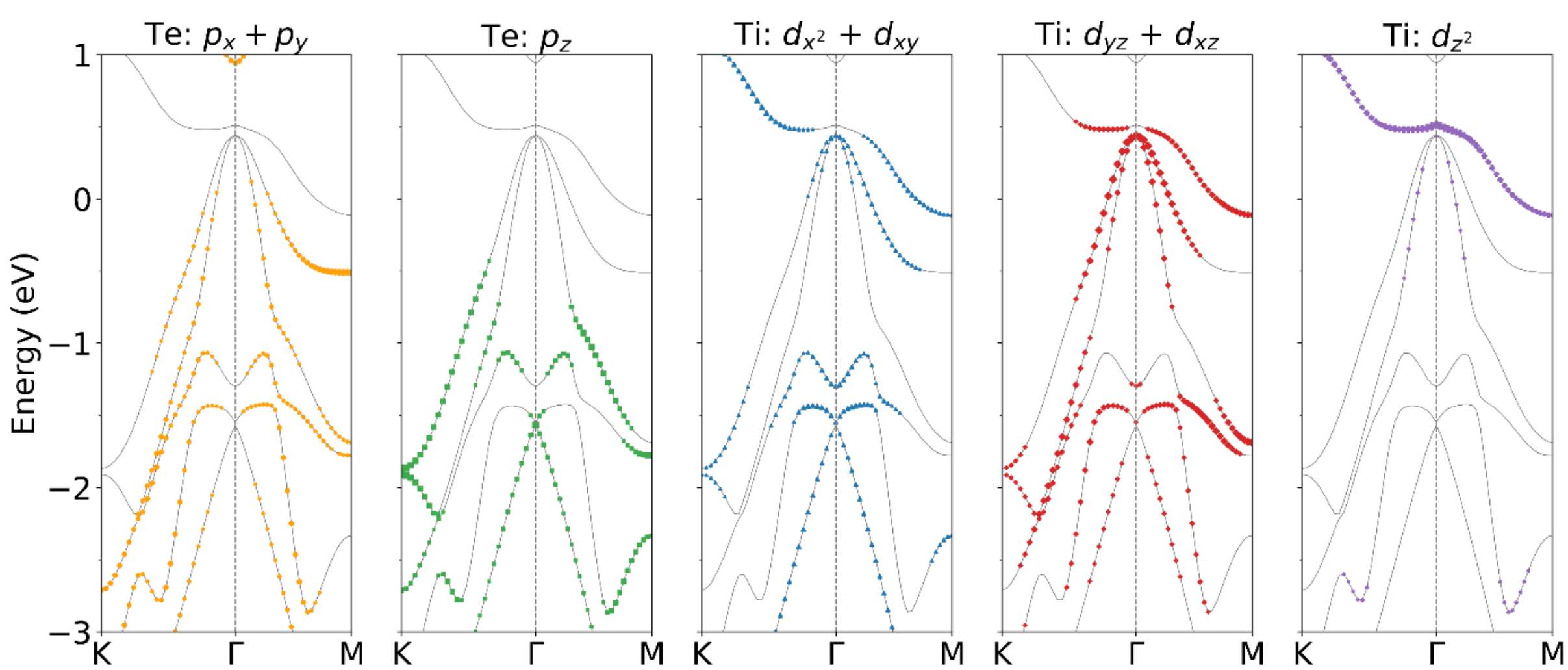

Figure S4. Orbital projected band structure for freestanding 1-TL $TiTe_2$.

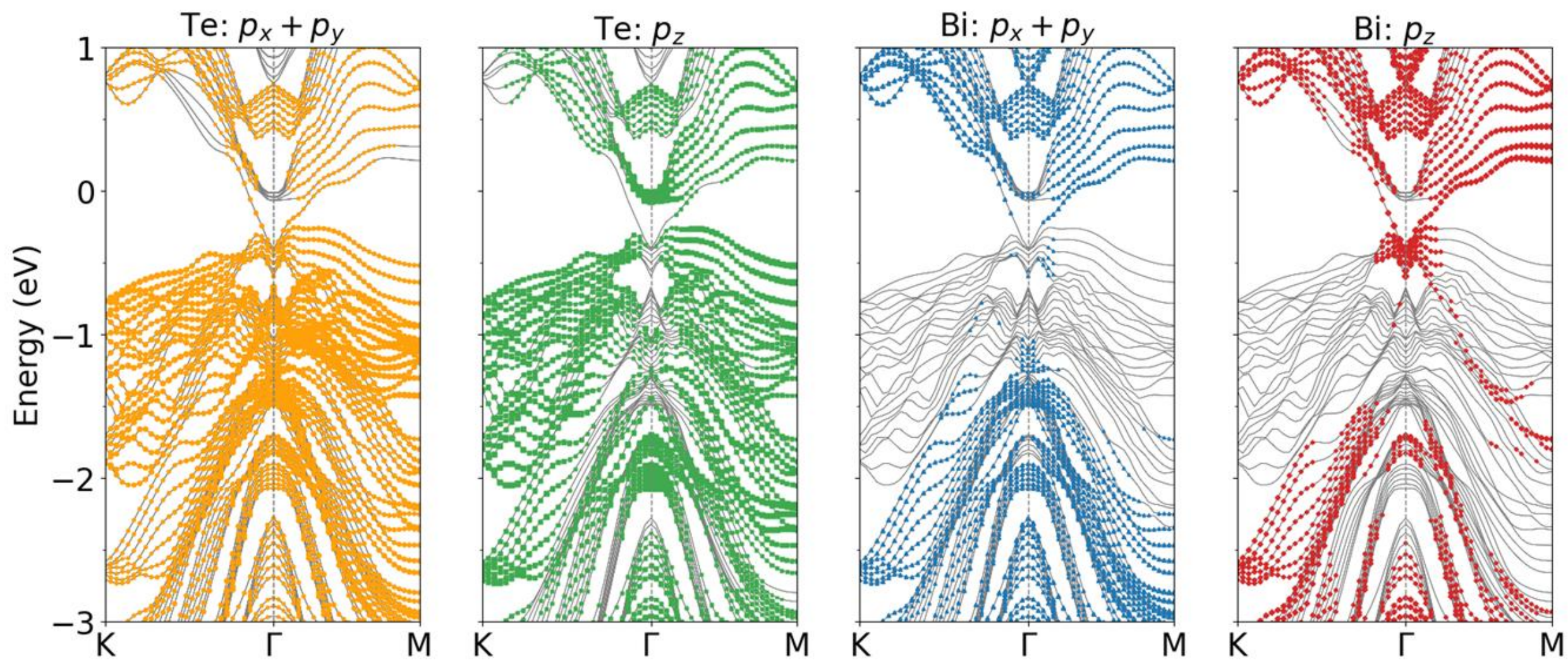

Figure S5. Orbital projected band structure for freestanding 7-QL Bi_2Te_3.

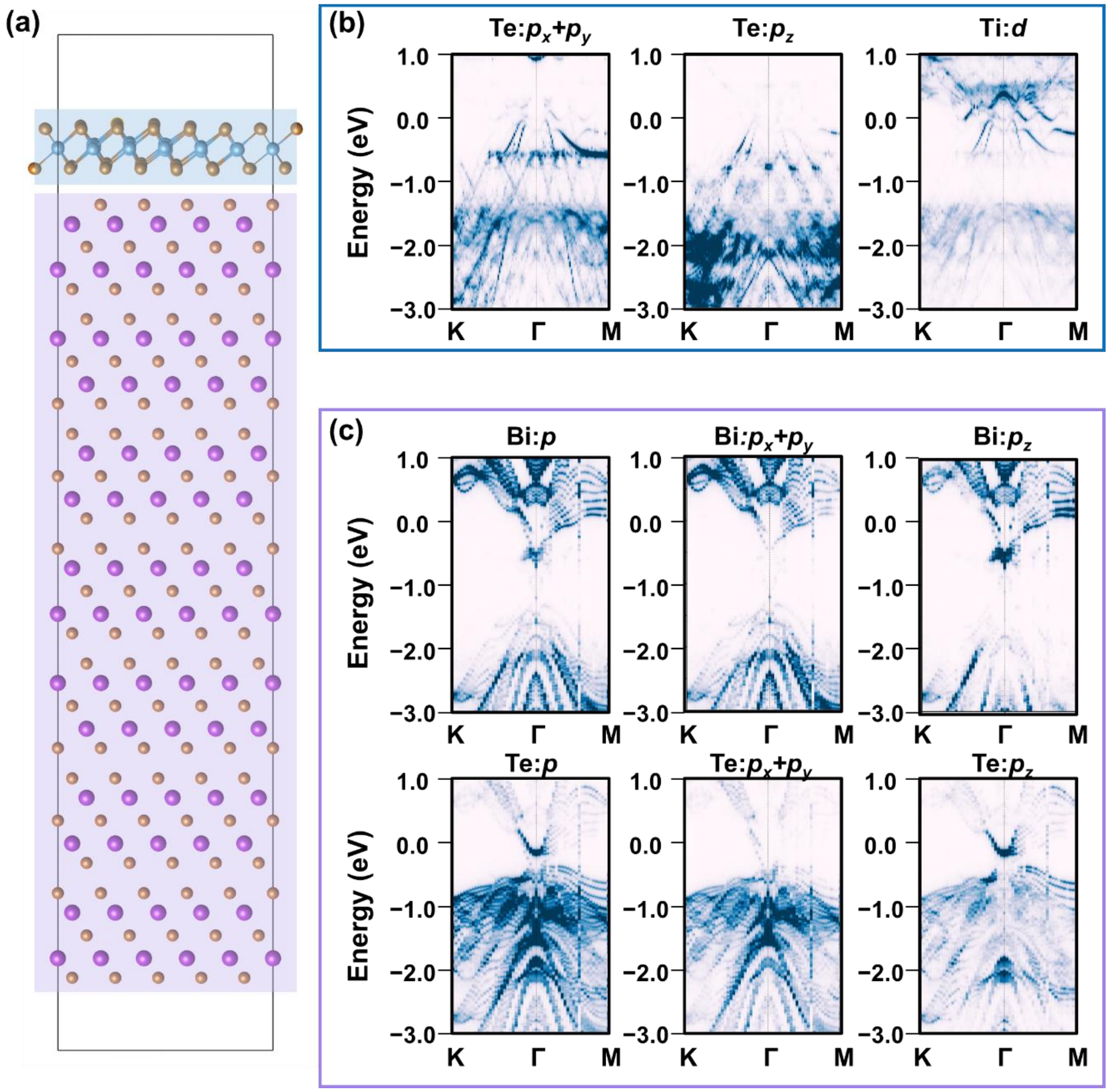

Figure S6. (a) Atomic structure of 1-TL $TiTe_2$ on 7-QL Bi_2Te_3. (b) Orbital projected band structure for $TiTe_2$ overlayer. (c) Orbital projected band structure for Bi_2Te_3 substrate.

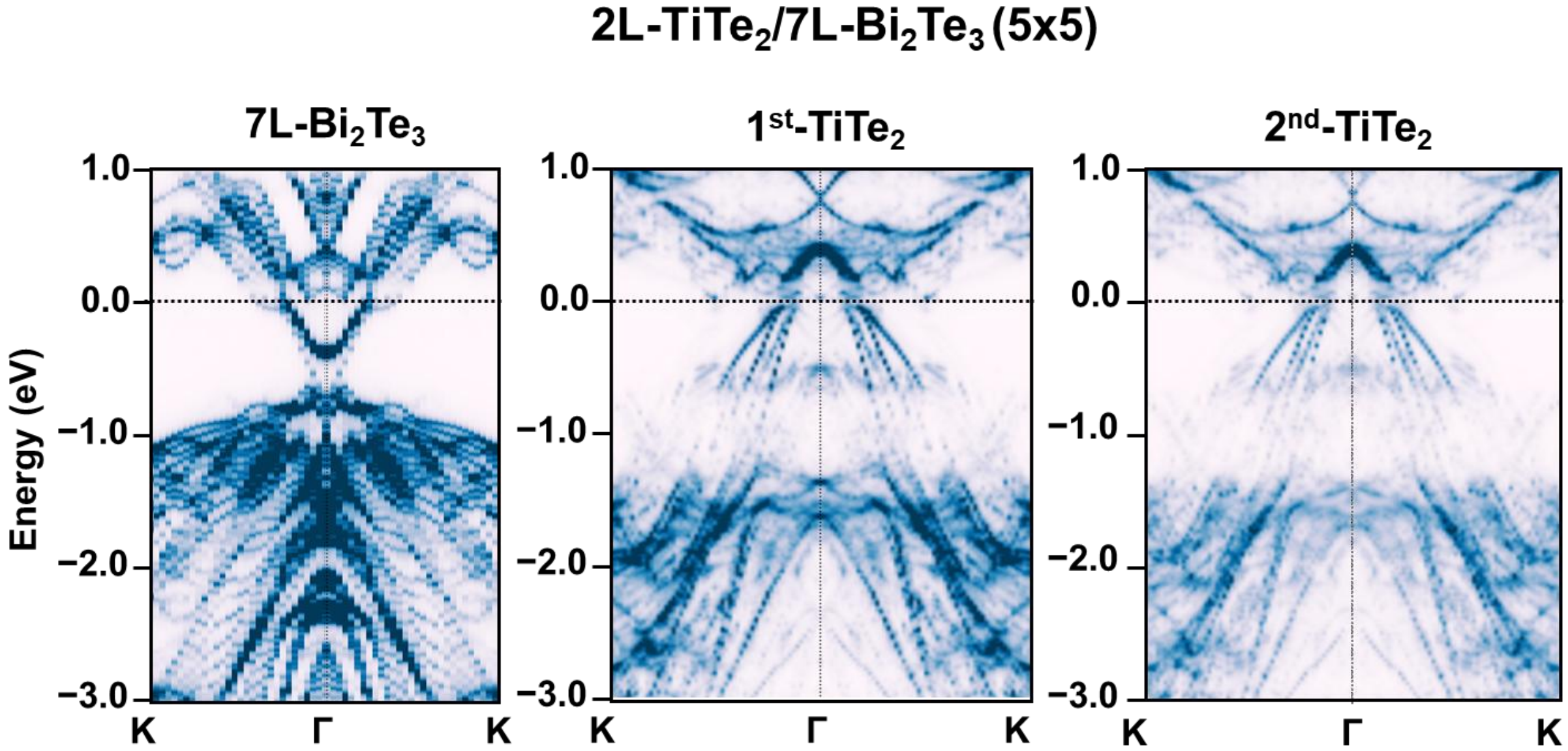

Figure S7. Layer-projected band structure for 2-TL $TiTe_2$ on 7-QL Bi_2Te_3. The upper and interfacial $TiTe_2$ layers are denoted as 1st-$TiTe_2$ and 2nd-$TiTe_2$, respectively.

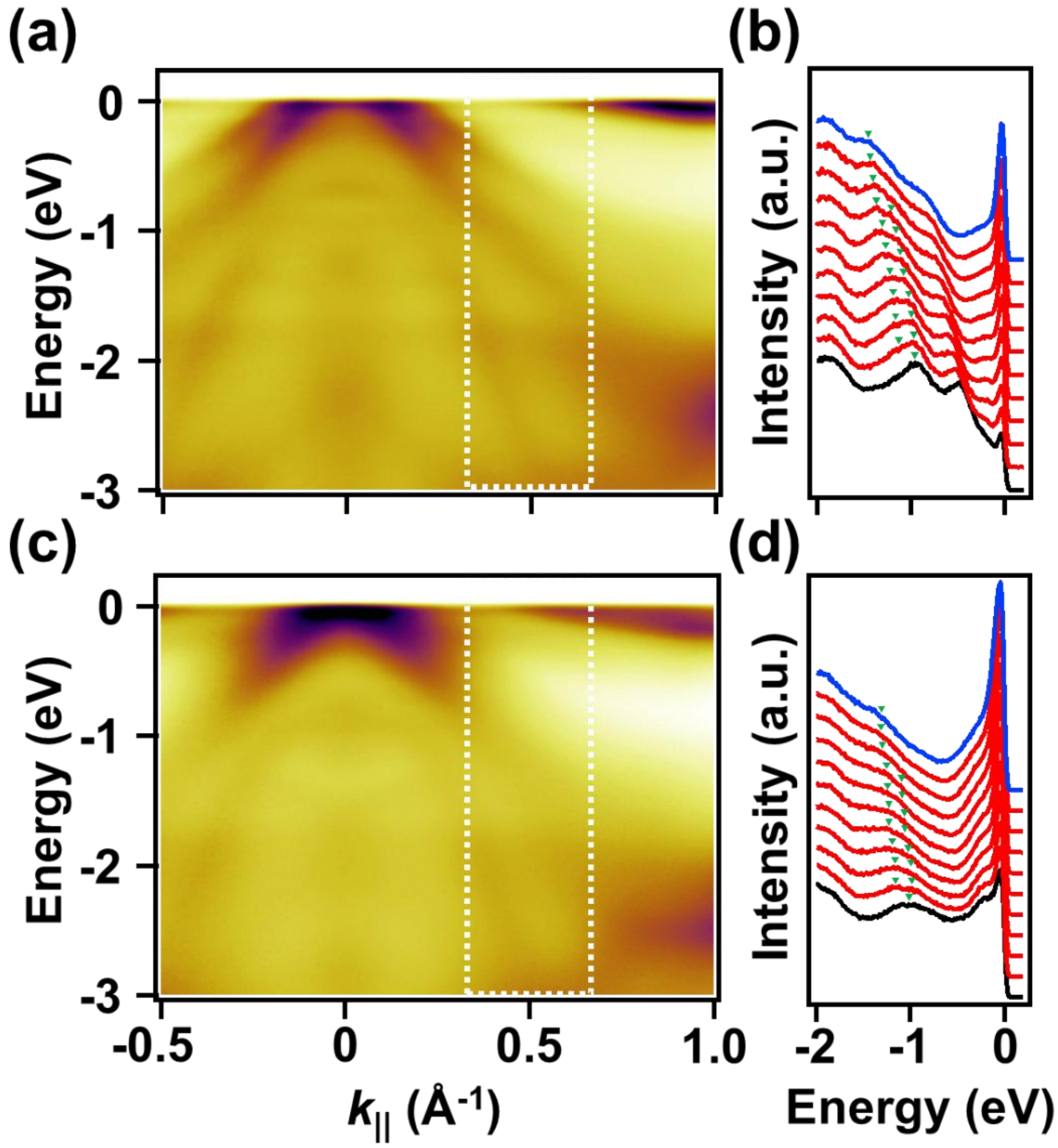

Figure S8. (a) ARPES map of 1-TL $TiTe_2$ on 7-QL Bi_2Te_3. (b) Energy distribution curves (EDCs) extracted from the original ARPES data at $k_{||}$ = 0.4–0.6 Å^{-1}, with a step size of 0.02 Å^{-1}. The selected momentum range is indicated by the white dashed lines in (a). (c), (d) same as (a), (b), respectively, but for 2-TL $TiTe_2$ on 7-QL Bi_2Te_3.

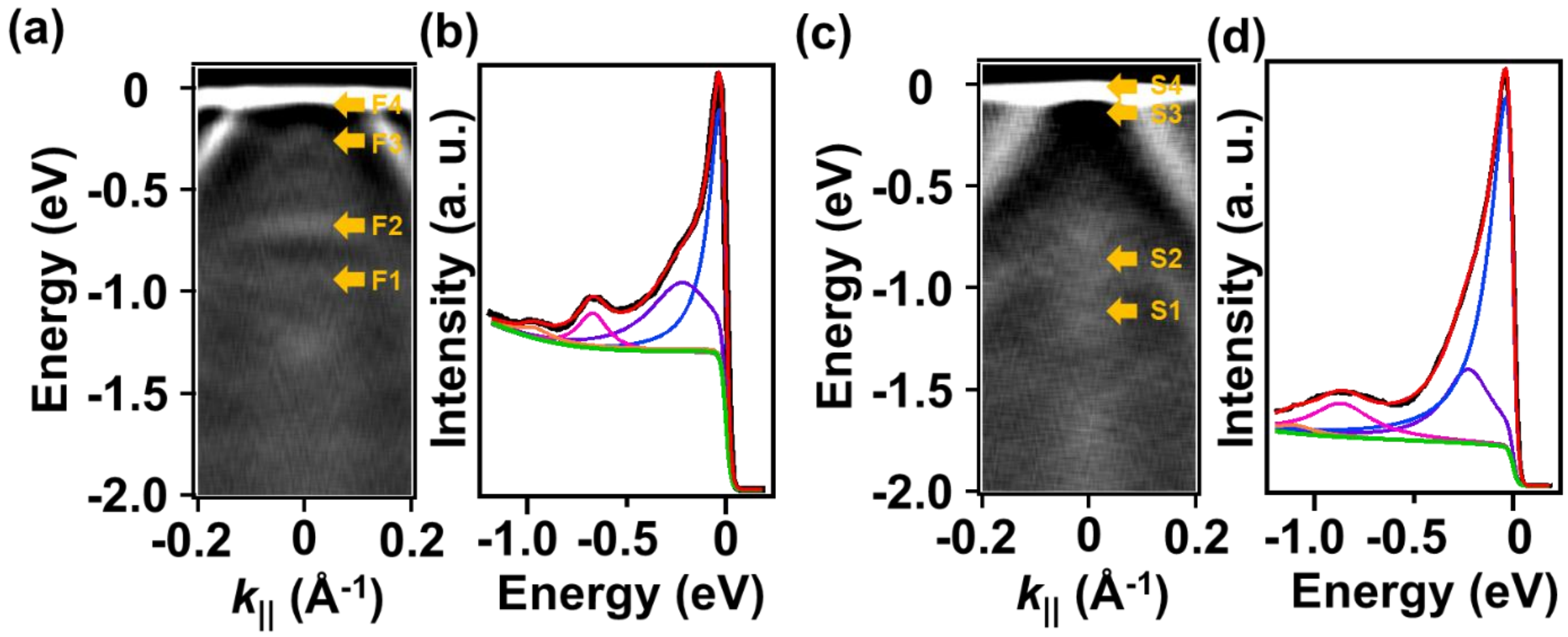

Figure S9. (a) Zoomed-in ARPES map of 1-TL $TiTe_2$ on 7-QL Bi_2Te_3. (b) Energy distribution curves extracted at the $\bar{\Gamma}$ and the corresponding fitting curves. (c), (d) same as (a), (b), respectively, but for 2-TL $TiTe_2$ on 7-QL Bi_2Te_3.

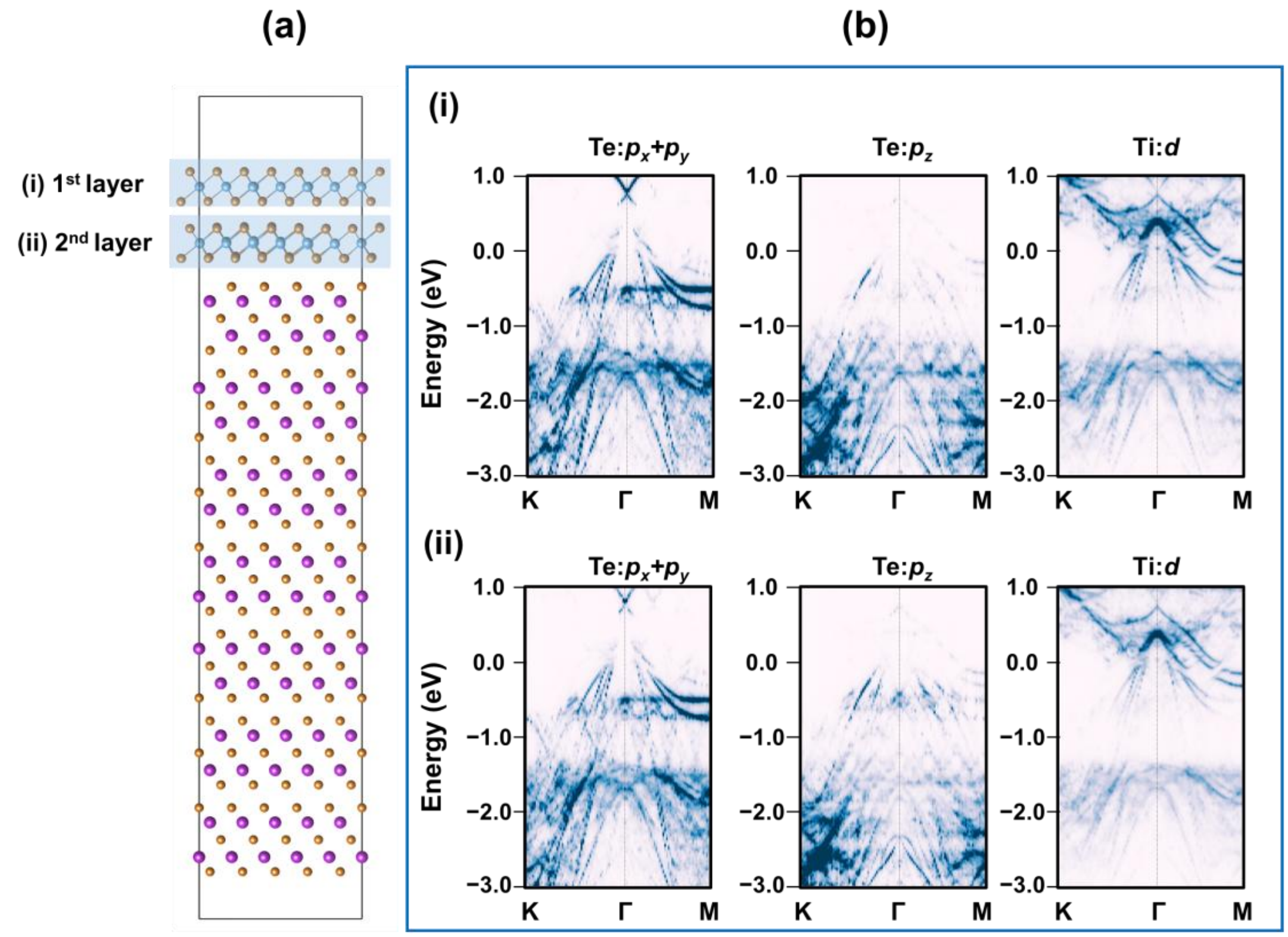

Figure S10. (a) Atomic structure of 2-TL $TiTe_2$ on 7-QL Bi_2Te_3. (b) Orbital projected band structure for (i) 1st-$TiTe_2$ and (ii) 2nd-$TiTe_2$.

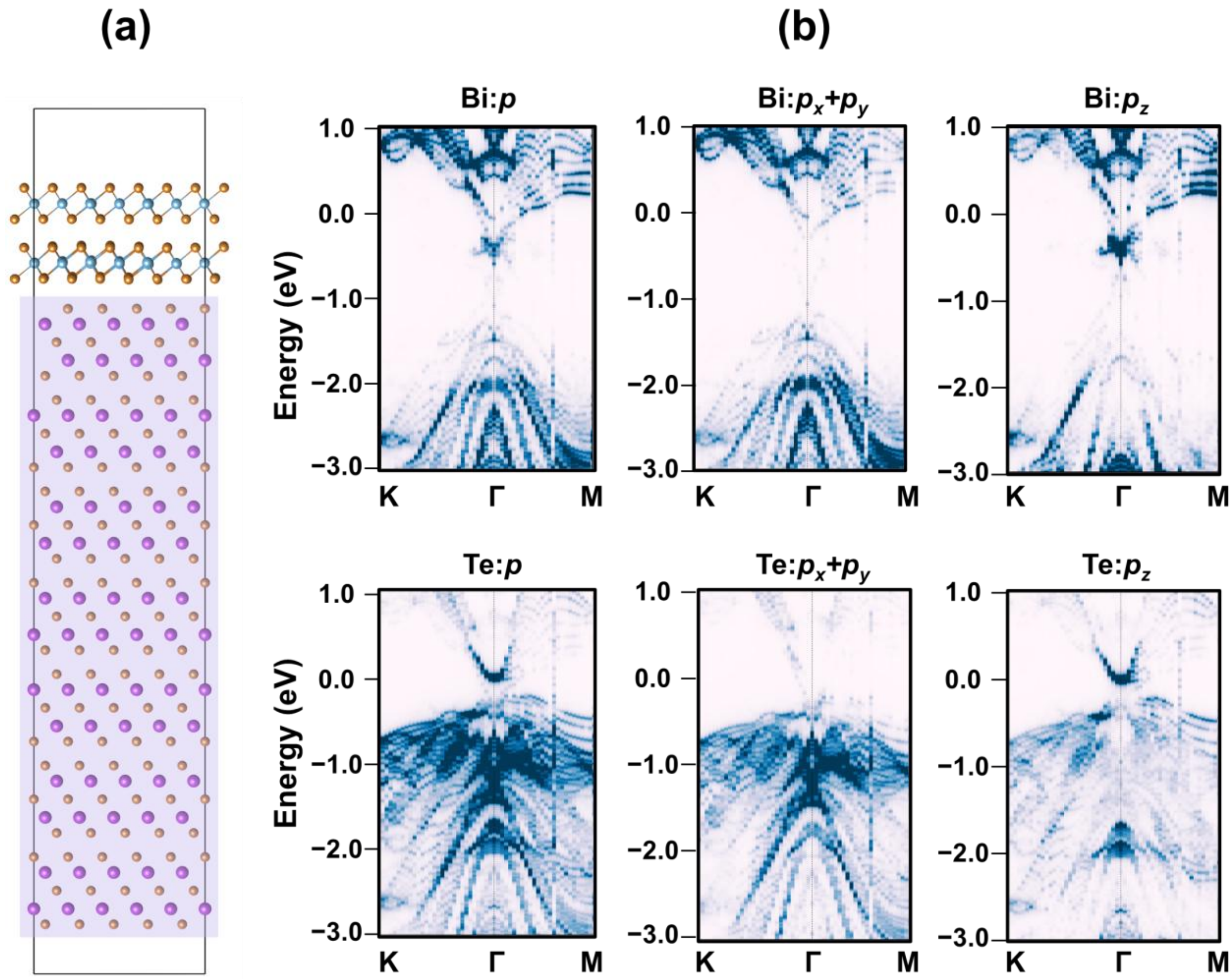

Figure S11. (a) Atomic structure of 2-TL $TiTe_2$ on 7-QL Bi_2Te_3. (b) Orbital projected band structure for Bi_2Te_3 (highlighted in purple region).

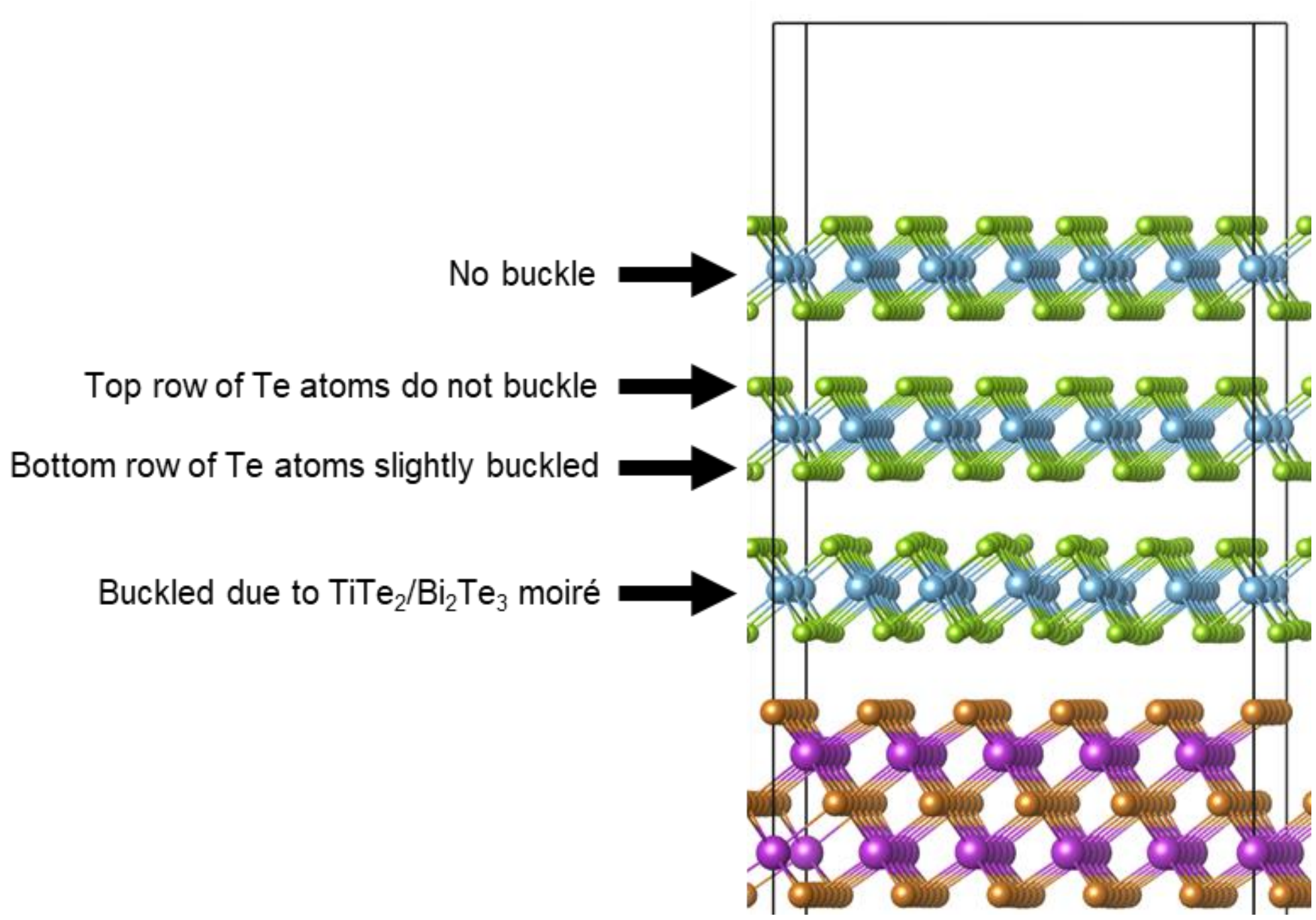

Figure S12. Optimized atomic structure of 3-TL $TiTe_2$ on 7-QL Bi_2Te_3.

Experimental details

Bilayer-graphene-terminated 6H-SiC(0001) substrates were prepared by repeated high-temperature annealing. Bi_2Te_3 films were grown by co-depositing Bi and Te from effusion cells under Te-rich conditions at 300 °C, with a growth rate of approximately 1 QL per 20 min. $TiTe_2$ layers were subsequently grown on Bi_2Te_3 by co-depositing Ti and Te at 280 °C. ARPES measurements were performed at 40 K using 21.2-eV photons for the $TiTe_2/Bi_2Te_3$ heterostructures and 60-eV photons at TPS Beamline 39A of the National Synchrotron Radiation Research Center for the $TiTe_2$/BLG films. Details of the film-thickness calibration, RHEED characterization, and film-uniformity checks are provided in the Supporting Figures S1 and S2.

Computational details.

The first-principles calculations were performed using the Vienna Ab-initio Simulation Package (VASP) [1,2]. The cutoff energy of 300 eV was used for the expansion of plane-wave basis set. The electron-ion interaction was treated by the projector-augmented-wave formalism [3]. The generalized gradient approximation within the scheme of Perdew−Burke−Ernzerhof (PBE) was employed for the exchange-correlation functional, while with the revised B86b vdW density functional (B86R) was used to address the relatively weak dispersion interaction [4,5]. The spin-orbit coupling (SOC) was included in all the calculations for electronic band structure. The band unfolding was conducted through the *easyunfold* package [6]. For the unfolded band structures, the spectral weights were projected onto the primitive $TiTe_2$ Brillouin zone to facilitate direct comparison with ARPES measurements.

The in-plane lattice constant of 3.72 Å and 4.428 Å was determined for $TiTe_2$ and Bi_2Te_3, respectively. To mitigate the strain imposed by the lattice mismatch between materials, the commensurate supercell with $TiTe_2$ (6 × 6) stacking on 7-QL Bi_2Te_3 (5 × 5) was implemented, see Fig. S6(a), leading to the minimized ~0.8% compressive strain exerted on the $TiTe_2$. The *k*-point meshes of 12 × 12 × 1 and 10 × 10 × 2 were used for the primitive monolayer $TiTe_2$ and bulk Bi_2Te_3. For the optimization of the supercell, only the Γ was taken into consideration. During structural relaxation, the bottom 6 QLs of Bi_2Te_3 were fixed, while the remaining atoms were fully relaxed.

Momentum-dependent EDC and peak-fitting analysis

Momentum-dependent EDCs were extracted from the original ARPES intensity maps along the selected momentum range $k_{\|}$ = 0.4–0.6 $Å^{-1}$ with a step size of 0.02 $Å^{-1}$ (Fig. S8). For the 2-TL heterostructure, the EDCs evolve from a single peak into a broadened/two-peak structure and then merge back into a single peak at larger momentum. The raw EDCs and phenomenological fitting results are shown in Fig. S9. The black curve represents the raw EDC, while the red curve shows the total fitting result. The green curve represents the smooth background including the Fermi-Dirac cutoff, and the other colored curves represent the individual Lorentzian peak contributions, each multiplied by the corresponding Fermi-Dirac distribution. The fitted peak components and background demonstrate that the weak features are present in the original intensity data rather than being generated solely by second-derivative processing.

Layer-resolved CDD definition

The layer-resolved CDD is defined as $\Delta\rho = \rho_{total} - \rho_{Bi_2Te_3} - \sum_{i=1}^{3} \rho_{i-\mathrm{TL\ TiTe_2}}$,where ρ_{total} is the charge density of the full heterostructure, $\rho_{Bi_2Te_3}$ is that of the isolated Bi_2Te_3 substrate, and $\rho_{i-\mathrm{TL\ TiTe_2}}$ represents the charge density of the individual $TiTe_2$ layers in the same atomic configuration as in the heterostructure. This layer-by-layer subtraction allows us to compare charge redistribution at different interfaces within the heterostructure, including the buried $TiTe_2$/Bi_2Te_3 heterointerface and the upper $TiTe_2$/$TiTe_2$ homointerfaces.

Moire-Modulated Redistribution at Interfaces

Because of the lattice mismatch between $TiTe_2$ and Bi_2Te_3, the buried heterointerface generates a moiré-modulated charge redistribution rather than a spatially uniform charge transfer. In Fig. 4 of the main text, although Interface B is a $TiTe_2$/$TiTe_2$ homointerface without an additional lattice mismatch, a residual moiré pattern remains visible. Its modulation amplitude is substantially reduced compared with Interface C, and its spatial distribution is approximately complementary to that at the buried heterointerface. This behavior indicates that the spatially varying perturbation generated at the $TiTe_2$/Bi_2Te_3 interface is transmitted into the second $TiTe_2$ layer through interlayer coupling.

Quantification of the substrate-induced perturbation in layer-resolved PDOS

To quantify how the substrate modifies the electronic states of the $TiTe_2$ film, we compared the layer-resolved projected density of states (PDOS) of the supported 3-TL $TiTe_2$ system with those of the corresponding freestanding 3-TL $TiTe_2$ slab. For the freestanding reference, the substrate was removed while retaining the $TiTe_2$ geometry, such that the intrinsic interlayer coupling within the 3-TL $TiTe_2$ film was preserved. The PDOS of the bottom, middle, and top $TiTe_2$ trilayers was evaluated separately for both the supported and freestanding systems, denoted

as $\mathrm{PDOS}_{\mathrm{i}}^{\mathrm{sub}}(E)$ and $\mathrm{PDOS}_{\mathrm{i}}^{\mathrm{free}}$, respectively. All PDOS spectra were aligned to the Fermi level, with $E_F = 0$ eV.

The substrate-induced PDOS variation for layer *i* (denoted as $\Delta\mathrm{PDOS}_i$) was defined as

$$\Delta\mathrm{PDOS}_{\mathrm{i}}(E) = \mathrm{PDOS}_{\mathrm{i}}^{\mathrm{sub}}(E) - \mathrm{PDOS}_{\mathrm{i}}^{\mathrm{free}}(E)$$

To quantify the magnitude of the substrate-induced electronic perturbation in each $TiTe_2$ layer, we further defined a normalized integrated PDOS difference, D_i,

$$D_i = \frac{\int_{E_1}^{E_F} \left|\mathrm{PDOS}_{\mathrm{i}}^{\mathrm{sub}}(E) - \mathrm{PDOS}_{\mathrm{i}}^{\mathrm{free}}(E)\right| \, dE}{\int_{E_1}^{E_F} \left[\mathrm{PDOS}_{\mathrm{i}}^{\mathrm{sub}}(E) + \mathrm{PDOS}_{\mathrm{i}}^{\mathrm{free}}(E)\right] \, dE}$$

Here, $D_i = 0$ corresponds to identical PDOS spectra, whereas larger values indicate a stronger substrate-induced modification. Because the denominator contains the sum of the two PDOS spectra, D_i is bounded between 0 and 1. It should be emphasized that D_i is a layer-specific normalized dissimilarity measure, evaluated independently for each $TiTe_2$ layer by comparing its supported and freestanding PDOS. Therefore, the values for different layers are not mutually normalized contributions to a common total, and their sum is not constrained to equal unity. A sum-to-unity normalization would instead describe the relative share of the total PDOS perturbation among the layers, which is a different quantity from the present D_i.

For the occupied states within 0.5 eV below the Fermi level, i.e., $E_1 = -0.5$ eV and $E_F = 0$, the calculated values are $D_{\mathrm{Bottom}} = 0.1278$, $D_{\mathrm{Middle}} = 0.1217$, $D_{\mathrm{Top}} = 0.0888$. Thus, the normalized PDOS difference decreases from the $TiTe_2$ layer adjacent to the substrate toward the outermost surface layer. This trend indicates that the substrate-induced modification of the near-E_F electronic

perturbation is strongest at the interface and becomes progressively weaker away from the substrate, consistent with a finite spatial extent of the interfacial electronic coupling.

Thickness calibration and film morphology

Before film growth, the deposition rates were calibrated using a quartz crystal microbalance (QCM) to determine the nominal thickness of the $TiTe_2$ and Bi_2Te_3 films. Following deposition, the surface structure was examined by RHEED. The RHEED patterns of the relevant films are shown in Fig. S1. The sharp streaks and absence of spotty or ring-like diffraction features are consistent with smooth, two-dimensional growth without appreciable three-dimensional island formation. As an independent electronic-structure check, the ARPES spectra of 7-QL Bi_2Te_3/BLG and 1-TL and 2-TL $TiTe_2$/BLG reproduce the characteristic band structures reported previously [references 23-28 in the main text]. The absence of obvious spectral features associated with coexisting film thicknesses further supports accurate nominal thickness control and a predominantly uniform film within the region probed by ARPES.

Supporting References

(1) Kresse, G.; Furthmüller, J. Efficient Iterative Schemes for *ab initio* Total-energy Calculations using a Plane-Wave Basis Set. Phys. Rev. B **1996**, 54, 11169.

(2) Kresse, G.; Furthmüller, J. Efficiency of Ab-Initio Total Energy Calculations for Metals and Semiconductors Using a Plane-Wave Basis Set. Comput. Mater. Sci. **1996**, 6, 15.

(3) Blöchl, P. E. Projector Augmented-Wave Method. Phys. Rev. B **1994**, 50, 17953.

(4) Perdew, J. P.; Burke, K.; Ernzerhof, M. Generalized Gradient Approximation Made Simple. Phys. Rev. Lett. **1996**, 77, 3865.

(5) Hamada, I. Van Der Waals Density Functional Made Accurate. Phys. Rev. B **2014**, 89, 121103.

(6) Zhu, B.; Kavanagh, S. R.; Scanlon, D. Easyunfold: A Python Package for Unfolding Electronic Band Structures. JOSS **2024**, 9, 5974.
